# MIND, Anti-Psychiatry, and the Case of the Mental Hygiene Movement’s ‘Discursive Transformation’

**DOI:** 10.1093/shm/hky096

**Published:** 2018-11-17

**Authors:** Jonathan Toms

**Keywords:** MIND, mental hygiene, social movements, anti-psychiatry

## Abstract

During the 1970s the National Association for Mental Health (NAMH) re-labelled itself MIND, becoming a rights-based organisation, critiquing psychiatry and emphasising patients’ citizenship. Its transformation has been coloured by attributions of the influence of anti-psychiatry. This article argues that the relevance of anti-psychiatry has been over-simplified. It examines MIND’s history as part of the psychiatric strategy known as mental hygiene. This movement’s agenda can be understood as paradigmatic of much that anti-psychiatry renounced. However, building on the sociologist Nick Crossley’s description of the interactional nature of Social Movement Organisations in the psychiatric field, this article shows that a ‘discursive transformation’ can be deduced in core elements of mental hygienist thinking. This transformation of discourse clearly *prefigured* important elements of anti-psychiatry, and also fed into MIND’s rights approach. But it must be appreciated on its own terms. Its distinctiveness under MIND is shown in its application to people with learning disabilities.

In 1970 the British mental health charity, the National Association for Mental Health (NAMH) announced a radical change of emphasis. NAMH was the leading charity organisation working in mental health at that time. It had been founded in 1946 as a major embodiment of what was known as the movement for mental hygiene, and an amalgamation of three of the movement’s constituent organisations whose origins stretched back into the early decades of the twentieth century. By the end of the 1960s, however, NAMH had acquired a staid image as a ‘twin-set-and-pearls’ establishment organisation working in close alliance with psychiatrists and the government. During the 1960s it had increasingly struggled to respond to rapid social change. Added to this a series of hospital scandals broke around this time, contributing to a sense of crisis in the psychiatric system.

NAMH responded by rebranding itself MIND and adopting the role of a consumer oriented organisation using rights-based terminology.^1^ Critiques of MIND’s activity during the 1970s and 1980s highlighted anti-psychiatry as an important influence and this has strongly influenced historiographical understanding. This article contends that the effect has been to occlude a fuller appreciation of transformations associated with the mental hygiene movement. Using newly available material, I build on my own work on the mental hygiene movement and combine this with the sociologist Nick Crossley’s analysis of social movement organisations (SMOs) in mental health, in order to reveal important themes that have been obscured.^2^

Delineations of anti-psychiatry have been diffuse. Yet, in their attribution to MIND, these delineations have commonly resolved into the view that MIND naively accepted anti-psychiatry’s debunking claims (along with the most radical versions of sociological ‘labelling’ theory), concluded that ‘mental illness does not exist’, and simply saw decarceration from psychiatric hospitals as the solution.^3^ It would be hard to deny that anti-psychiatry had some influence on MIND. For example, leading figures were especially influenced by Erving Goffman as well as the Italian psychiatrist Franco Basaglia’s radical alternative to mental hospitals.^4^ However, MIND did not deny the existence of mental illness, nor did it envisage decarceration as a straightforward solution to people’s troubles. Indeed during the late 1960s NAMH came under sustained attack from another strand of anti-psychiatry, the Church of Scientology, whose Citizens Commission on Human Rights was founded with Thomas Szasz. This conflict continued with MIND.^5^ On the other hand, MIND was supportive of the Mental Patients Union (MPU) which was set up in 1973 and was strongly influenced by anti-psychiatry, as well as Marxist thinking. MIND gave space in its magazine *MIND OUT* for MPU news and views. But it soon came under criticism from elements of the MPU and associated groups who considered it to be insufficiently representative of service users.^6^

The impact of anti-psychiatry is therefore complex. Its relevance to MIND and the mental hygiene movement, I argue, lies elsewhere, in its ideological underpinnings and core features. In his *The Deviant Imagination*, Geoffrey Pearson provided an early and sophisticated attempt, to unpack its assumptions and imperatives. He noted that:

One of the most important, if not *the* most important, developments in the field of deviance has been the ‘medicalisation’ and ‘psychiatricisation’ of social problems: crime, it is said, is an illness; youthful unrest is a maturational phase; political dissent is the result of personality quirks of ‘mindless militants’; a poor employment record flows from a disorder of character; poor families, or ‘problem families’ are low on something called ‘interpersonal maturity’. Some critics will say that the worst thing to happen to deviance theory was medicine.^7^

For Pearson, anti-psychiatry offered a ‘*politics of socialisation*’ that exposed how under urban-industrial society ‘the requirements of normality, conformity and reasonableness—in a word “mental health”’ distorted human consciousness. Anti-psychiatry emphasised human freedom and choice, at the same time as it problematised authority and coercion. Its alternative programme has been summarised by Digby Tantam: ‘Overcoming alienation by means of alternative therapy, self-help, the abolition of the distinction between professional and patient, and the levelling of hierarchy …’.^8^

This article is divided into three sections. In the first, I summarise Nick Crossley’s rendition of the trajectory of the mental hygiene movement and the impact of anti-psychiatry. In the next, I offer my own description of the mental hygiene movement and NAMH. This largely supports Crossley’s, but draws attention to certain significant omissions and aims to deepen the analysis. In the third section, however, I explain how leading principles of mental hygienist therapeutic theory manifested a ‘discursive transformation’. These can be understood as prefigurative of important elements in anti-psychiatry, but they must also, I argue, be recognised as offering a distinct conceptualisation of mental health and ‘disorder’. In the final section I show how these principles were brought into an unstable coalition with wider elements of social critique under MIND.

## Crossley’s Schema in Relation to the Mental Hygiene Movement, and Anti-Psychiatry

Crossley’s analysis of post-war SMOs focused on their dynamic and interactional relation, and the importance of discursive formation, diffusion and transformation.^9^ It recognised the importance of the mental hygiene movement, as well as that of anti-psychiatry and civil rights.

Crossley noted that the mental hygiene movement emerged in Britain between the two World Wars and remained an important institutionalised movement throughout the post-war years until the turn of the 1970s.^10^ He focused on NAMH, and its interwar precursor bodies: the Central Association for Mental Welfare (CAMW), the National Council for Mental Hygiene (NCMH) and the Child Guidance Council (CGC). For Crossley the mental hygiene movement could be characterised as both ‘progressive’ and ‘conservative’. In his view the discourse of mental hygiene, advocated by NAMH and its predecessors expressed a middle-class bias along with associated moral and gender biases that ‘tended to preclude the possibility of debate (and thus democracy in psychiatry) by virtue of their appeal to a realm of pre-established scientific fact’.^11^ By this, Crossley appears to mean the restriction of debate within the field of psychiatry.

But for Crossley the mental hygiene movement also expressed a ‘progressive’ aspect. He noted, for instance, that the mental hygiene movement placed a greater focus on ‘environmentalism’ challenging the attribution of mental disorders exclusively to organic causes, common among British psychiatrists. This is a shift that was particularly related to psychiatrists’ and psychologists’ experience of the Great War.^12^ However, Crossley’s main emphasis was on the movement’s switch in emphasis from the terminology of ‘sanity’ and ‘insanity’ to ‘mental health’ and ‘mental illness’. It promoted the full development of personality in the name of ‘mental health’ and this involved a nationally organised system of education, detection and early treatment. In the process, the movement sought a shift away from ‘the old asylum system with its punitive, poor law associations’ to a service oriented towards the community, out-patients’ clinics and child guidance services.^13^ Inherent in this was the promotion of associated professions of child psychiatry, psychology and psychiatric social work.

Crossley convincingly argues that while the mental hygiene movement’s ‘goal of improving mental health and reorienting services away from illness towards health’ was not fully accepted in psychiatry, or wider society, it nevertheless amounted to a ‘paradigm shift’. The mental hygiene movement shifted the overall conceptualisation and orientation of the psychiatric system.^14^ From the interwar period, the government accepted the mental hygienist call for early treatment and the associated need for attitudinal changes in the wider public.^15^

However, one of the ironic effects was to contribute both to an ‘elevation of expectations’ that remained unmet, and a wider questioning and debate about the psychiatric system. In Crossley’s terminology, this created ‘strain’ within the ‘psychiatric field of contestation’ and encouraged the emergence and engagement of other SMOs within the psychiatric field of contention; first the National Council for Civil Liberties (NCCL) during the 1950s, and later an ‘anti-psychiatry’ movement.^16^

The NCCL was the earliest post-war SMO to enter the field in antagonism with psychiatry and the mental hygiene movement. Crossley notes that the well-established civil rights discourse provided the organisation, mobilisation and ‘discursive elaboration’ required to turn ‘strain’ within the field of psychiatric contestation into active campaigning for reform.^17^ NAMH adopted a ‘conservative’ stance, ‘reducing critique to ignorance’ of psychiatric expertise.^18^ Crossley claims that ‘there was little that was new in these debates’ but that the later anti-psychiatry movement set in train a ‘paradigm shift in the wider campaigning culture’. After anti-psychiatry ‘nobody could carry on as before’.^19^ The mental hygiene discourse appeared outmoded. NAMH could not shift its stance to the extent of adopting the agenda of anti-psychiatry. Instead it ‘embraced a civil rights discourse’.^20^

## Mental Hygiene: ‘Adjustment’, ‘Familial Relations’ and Democracy

As Crossley argues, the mental hygiene discourse grounded its agenda on a claim to established scientific fact. Through this, it foreclosed debate and therefore ruled out ‘democracy in psychiatry’. This contention needs, however, to be explicitly integrated with the fact that the mental hygienist agenda also actively operated against any wider advocacy of egalitarian participatory democracy in society. The mental hygienist theorisation of the need for ‘mental adjustment’ and associated attention to familial authority relations appears central to this aspect of the mental hygiene discourse. I elaborate this below.

Two interacting theoretical discourses can be isolated as primarily informing the movement when it became institutionally established in Britain between the wars: a theory of instincts drawn from ‘the new psychology’ of the early twentieth century,^21^ and psychoanalysis along with its various popular reformulations. The first emphasised that primitive instincts and their accompanying emotions were basic impulses of human behaviour.^22^ The second attended to unconscious motivations within the mind. In combination these theories propounded the need to deal with the emotional aspects of the mind. Emotions underlay humans’ dynamic growth and ‘adjustment’. They could not simply be suppressed. The ability to understand how emotional experience was a necessary component of mental development was conceived as rational, scientific and more humane. The mental hygiene movement explicitly promulgated its scientific credentials. As the psychologist Cyril Burt, a member of the NCMH, stated it in an early 1930s radio broadcast:

Modern civilization is based upon science; and it is our belief that, if that civilization is to continue, scientific thinking must be applied to man as well as to inanimate nature. The methods that have revolutionised agriculture, industry, medicine and war, must be adopted for the study of ourselves—of the individual, the family, the nation and the race.^23^

Such a scientific approach was considered, by definition, to combine the potential for greater human happiness and greater societal efficiency. The aim was that a person should ‘learn how to govern his instincts so as to avoid conflict in his own mind and conflict with his neighbours’.^24^ While contemporary Western society was, for mental hygienists, a manifestation of progress, it also carried with it possibilities of destabilisation and even regression. Mental hygienists concerned themselves with societal cooperation and regulation in the face of concerns about the effects of industrialisation, commercialisation, increasing urbanisation and the emergence of popular politics. But while mental hygienists recognised the stress and complex social pressures that modernity had brought with it, their main focus was on some people’s failure, as they saw it, to adjust to these appropriately.^25^

Mental hygienists were concerned to avoid rampant egoists or atomistic individuals on the one hand, and suggestible sheep following the herd on the other.^26^ Adequate adjustment required an acceptable balance between these two ‘maladjusted’ extremes. A main support for this was the movement’s conceptualisation of ‘mental deficiency’. Along with certain categories of people certified insane, mental deficiency performed the role of dramatically highlighting what mental hygienists saw as a substantial negative hereditarian impact on social regulation, citizenship and social progress. As Hugh Crichton Miller, the director of the Tavistock Clinic put it, ‘A hereditary deficiency of grey matter makes a good adjustment impossible’.^27^ These people’s close association, by mental hygienists, with an array of social problems, was coupled with representations of them as either suggestible and easily lead, or egoistic and without restraint. For everybody else, mental hygienists considered the preeminent locus of adjustment to lie in the family.

In the early 1980s the sociologist David Armstrong provided an influential account of how, in the early decades of the twentieth century, ‘the medical gaze’ shifted from locating disease in bodies and specific locations to tracking it in the interstices between bodies. He detected an extension of this gaze to mental troubles, from around the Second World War. ‘Mental instabilities’ began to be conceived as actually manifest in relationships themselves.^28^ Reconfigured approaches along these lines, emerging in the discipline of psychoanalysis, were particularly closely associated with mental hygiene. The psychoanalyst Joseph Schwartz has described these as the beginnings of a ‘paradigm shift’ in psychoanalysis. The classical understanding of an internal psyche seeking tension reduction through the satisfaction of desires was transformed by the gradual elaboration of a theory of personality conceived in terms of the need for human relationships.^29^ These transformations had wide ramifications. For example, Chris Millard has recently traced them to a shift in the psychological conceptualisation and treatment of self-harm in Britain.^30^

For mental hygienists the family lay at the root of social order and stability. Maurice Craig, the President of the NCMH, wrote, ‘Mental hygiene has its beginning in the home. Family life is the basis of all social order, and everything must be done to make the units which compose it harmonious and stable’.^31^ The psychiatrist and mental hygienist, R. D. Gillespie, wrote that, ‘the family environment is literally of immense importance for psychopathology and the understanding of people whether as healthy beings or as morbid, fearful, hysterical individuals’.^32^ Mental hygienists foregrounded, in particular here, the importance of personal relationships of authority. It was through these that the movement sought the development of a stable, integrated self, safely situated in a stable, integrated, and yet progressive, society. Emanuel Miller, a leading psychoanalytic psychiatrist in child guidance, wrote of the family’s intimate relations of authority that, ‘It is the gentleness, the regularity and the firmness of these rules which give to the young human being the first pattern of order and law’.^33^ A child needed to develop through personal relations of authority without being made either ‘ultra-suggestible’ or ‘a rebel against all authority’.^34^

Mental hygienists’ rendition of the modern individual was of someone who, through adequate mental development, had become rationally marshalled, and was able to take this balanced mental authority with them into the wider social order. Internally balanced and adjusted, it would readily acquiesce in the modern authority relations necessary to the balance and progress of wider society.

The mental hygiene agenda cut across notions of the body politic comprising a social contract, and addressed both the extent and contents of citizenship.^35^ Mental hygienists’ concern for the progressive development of both individual minds and society entailed more emphasis on a differentiation of social status and function according to mental ability and personality integration, and less emphasis on the coming together of equals implied by the notion of a social contract. Many mental hygienists explicitly expounded the need to accord social status with intellectual capacity.^36^ Some, such as Cyril Burt and Hugh Crichton Miller explicitly questioned whether political democracy should be amended on the basis of individuals’ intellectual capacity.^37^

Similarly, mental hygienists’ concentration on familial authority relations shaped, for example, their attention to the problem of the ‘misfit’ in employment. They believed vocational misfits could be the result of an intelligence or temperament wrongly placed, or be the product of earlier faulty relations in the family creating emotional maladjustments. The ‘misfit’ was closely associated with industrial problems. Implicitly focusing attention on workers rather than employers, Maurice Craig, propounded that, ‘Maladjustments of individuals cause great social and industrial problems, and it is necessary to understand these both in the interests of the manual worker and those who employ them’.^38^ Gillespie connected both the psychological interpretation of square pegs in round holes and the conviction that mental maladjustment resulted from faulty authority relations in the family, with ‘industrial problems’ and political dissent.

He wrote that,

from the collective aspect a group containing a large proportion of defective and emotionally unstable persons is a menace to industrial peace. It has been shown that even among normal people in employment who have monotonous tasks to perform hour after hour, day after day, mental activity consists mainly in discontented thoughts, rumination over supposed injustice regarding pay and promotion and other conditions of work. A state of mind of this sort is fertile soil for the agitator of political and economic discord.^39^

Likewise, other mental hygienists attributed industrial strikes to the consequences of faulty parental authority among the working classes and similar reasoning was applied to wider political conflict.^40^

Crossley maintains that mental hygienists’ claim to scientific authority foreclosed debate and inhibited ‘democracy in psychiatry’. But, clearly as well, the intellectual and class bias inherent in much mental hygienist theorising operated against democratic and egalitarian claims within wider society. It is clear too, that this mental hygienist programme was a forthright rendition of much that anti-psychiatry renounced.

Crossley maintains that there was nevertheless a ‘progressive’ aspect to the mental hygiene agenda: its promotion of mental health and the early treatment of mental disorders. Through this the movement influentially promoted changes to the form of psychiatric organisation, with clinics and services based in, and oriented towards the community, and the establishment of associated professions. Certainly, mental hygienists were successful in gaining governmental support and Crossley’s assertion that the mental hygiene movement’s agenda resulted in a paradigm shift is persuasive.

Mental hygienists succeeded in influencing the 1926 Royal Commission on Mental Disorder to the extent that the objectives which informed the Commission were accepted as public health, mental hygiene, national efficiency and social reconstruction.^41^ The keynote of the future’, asserted the Commission, ‘should be prevention and treatment’.^42^ Yet the voluntary patient status that was proposed by the Commission (and enacted in limited fashion under the 1930 Mental Health Act) was not intended as recognition of the freedom of patients to decide whether to enter or leave hospital in their own interests. As Clive Unsworth noted, it served to reduce the penal character of entry to mental hospital at the same time as it promoted a notion of social responsibility to submit to public health measures necessary to the interests of the community as a whole.^43^ The hope was to encourage people suffering mental stress to submit to early treatment.

Mental hygienists considered child guidance clinics central to providing comprehensive mental hygiene for the community.^44^ Yet child guidance clinics discriminated against treating children considered ‘mentally deficient’. A judgement that they were ‘deficient in intelligence’ also deemed them unable to benefit from the psychological treatment others received for emotional difficulties. Child guidance also commonly expressed a gender bias. For example:

The mother is the centre-piece of the family when it is a matter of describing the family neurosis, but it is to the father we must look when it comes to prognosis and treatment. He more than anyone else will have an effective influence on his wife and children if we can release his normal masculine impulses. He will then encourage what is normal in his wife, but even more important is the influence which he will bring to bear directly on his children. He will make his daughter proud and happy in her femininity and give his sons an example they can follow with pride.^45^

Nevertheless, as Crossley emphasises, the mental hygienist attempt to shift the form of psychiatric organisation, from isolated mental hospitals to a nationally integrated system that included child guidance and out-patient clinics, was significant among the ‘successive rounds of reform of psychiatry in the 1930s and 1950s’. He is surely right, as well, that an unintended effect was to elevate expectations which remained unmet, and promote wider debate and criticism of the psychiatric system. This lead to the entry of the National Council for Civil Liberties (NCCL) into the ‘psychiatric field’, and the later emergence of ‘anti-psychiatry’.^46^

I concentrate in the next section, however, on how a ‘discursive transformation’ developed in the *content* of the forms of psychiatric organisation that the mental hygiene movement promoted. The therapeutic principles developed by the mental hygiene movement had, as we have seen, foregrounded the importance of familial relations of authority. Within this they had prioritised emotional experience and expression as crucial components of ‘mental adjustment’. Emotionality needed to be understood, nurtured and accommodated in the interests of a concept of ‘mental health’ conceived in terms of good citizenship and ‘social efficiency’. An important aspect of this nurture and development was commonly referred to as a need for ‘emotional security’. I explain how an understanding of the impact of this terminology enables an appreciation of the discursive transformation that appears to have taken place at the level of therapeutic theory, and (to a certain extent) practice.

## Mental Hygienist Therapeutic Principles and ‘Discursive Transformation’

Speaking at a 1957 NAMH conference on ‘The Maladjusted Child’, the psychiatrist and leading mental hygienist, Kenneth Soddy, told his audience that a doctor friend of his had sent him a cutting from the ‘New Yorker’:

The picture shows a psychiatrist talking to the mother and very glum-looking child. Underneath is the caption: ‘Mrs Minton, there’s no such thing as a bad boy. Hostile perhaps. Aggressive, recalcitrant, destructive, even sadistic. But not *bad*.’^47^

But Soddy did not disavow this caricature. In fact he remarked, ‘Now I happen to believe that so firmly that I have pleasure in passing it on to you today.’ The root of the caricature and of Soddy’s refusal to distance himself from it, was the mental hygienist concern with ‘emotional security’ as the central factor of satisfactory mental adjustment from infancy. Modern parents needed to recognise that their authority, in its manifestation as care and discipline, must be tailored to the child’s individuality and its need to pass successfully through maturational stages. Parents must listen to emotionality in terms of the developmental stages that it displayed, and tailor their authority accordingly. Behavioural issues were often ascribed, by mental hygienists, to emotionally insensitive relationships, and understood as inappropriate but emotionally communicative responses. A rationally marshalled and emotionally integrated individual could only satisfactorily develop if the child experienced emotionally secure relationships in the family. So, for example, as the psychoanalyst Mary Barkas put it in 1932, ‘With this emotional relationship and arising from it comes the dawning of a sense of guilt, and it depends largely upon the adult whether this relationship is wisely or unwisely utilised’.^48^ And as the psychiatrist Denis Martin put it in the 1960s, ‘the emotional security of the child in terms of being consistently loved, accepted and allowed to grow seems to be the crucial factor’.^49^

There is an emphasis on cognitive development in this characterisation that is important. Mental hygienists had drawn a line of distinction marking off those people considered constitutionally unable to benefit properly from this intimate and receptive family environment.^50^ The basis from which the concern for emotional security had developed was a belief in progress, understood as the twin development of societies and of individual minds. People designated mentally deficient were considered the failures of this process, for them a mental deficiency system had been constructed that used different conceptions of care and control.

Outside of the mental deficiency system, however, the mental hygienist concentration on ‘emotional security’ provoked a cluster of responses that acted both to undermine the foundations of the mental hygiene strategy, and inform the emergence of the rights approach of MIND. A concise way into the issues is to look at the mental hygiene movement’s response to the Curtis Committee on the Care of Children Deprived of a Normal Home. This was set up by the government in 1945 in response to concerns about children’s institutional care largely raised by the wartime evacuation.

Early in the war the CAMW, NCMH and CGC, had integrated their work and become involved in the evacuation measures. They set up a nationally coordinated scheme to oversee social and psychological issues. Working with central and local government, they helped organise and provide advice for wartime hostels, residential nurseries and already existing Public Assistance Homes. On the basis of this work they compiled a memorandum of evidence to the Curtis Committee.^51^

This report made clear that the aim of all childcare must be to enable the child to achieve a healthy maturity. In accordance with all mental hygienist child care theorising, it maintained that any upbringing outside the biological family was necessarily handicapping to the child. It therefore recommended that all institutional settings must aim to compensate for this as much as possible. A child should be provided with a close, continuous and trusting relationship with a caring adult. Day to day life should be in small groups of mixed ages, with the opportunity for freedom and diversity in occupations, possessions and leisure.

The promotion of these principles was accompanied by a critique of prevailing institutional provision for children. The report exposed the fact that life in large groups promoted disturbances in development and mental health ranging from bed-wetting, to emotional disturbance and anti-social behaviour. Hospital-like regimes were criticised for their over-concern with personal hygiene, cleanliness and efficiency, as well as an excessive requirement for order and control. Most regimes were considered insensitive to the needs of children’s emotional development, thus distorting them and creating maladjustment. The report maintained that ‘culture is not imposed by lessons … but by the feelers a child puts out to draw them into himself’. ‘Children, like adults’, it remarked, ‘need to be able to reject or turn down what is offered them.’ Staff’s sensitivity to the dynamics of children’s emotional lives therefore came under scrutiny. They should avoid arbitrary authority or placing an exaggerated importance on their position. There was an implicit concern here to diminish differentials in status between children and staff, and also to some extent between staff themselves. The centre of practice was to be an appreciation of children’s on-going need for affection and emotional security. The report stated bluntly that it was only children who had become passively conforming and isolated from secure emotional relationships who appeared to accept typical institutional life.

The mental hygiene movement did not, however, consider the principles outlined in this report to be relevant to mentally deficient children in institutions. However, during the early 1950s psychologists later associated with NAMH began research that applied mental hygienist ideas on emotional development and institutionalisation to detained mentally deficient people who were categorised ‘feebleminded’.^52^ Crossley’s schema suggests that the intrusion of a civil rights discourse, through the campaigning of the NCCL over the mental deficiency system in the early 1950s, created ‘strain’ in the mental health field. This appears to have been one consequence. The research had marginal impact at this time, but it nevertheless highlights the interactional nature of SMOs that Crossley emphasised. In fact, although he noted that NAMH’s public response to NCCL campaigning was hostile, it shows that beneath the surface deeper engagements were taking place.^53^ The psychologist Jack Tizard went further in implicitly challenging the mental hygienist psychotherapeutically inspired principles of child development, in what became known as the Brooklands experiment, carried out at the end of the 1950s. He, and his colleagues, applied to children termed ‘imbecile’ the principles of childcare that the Curtis Committee had made the foundations of post-war policy for other looked-after children.^54^ While NAMH’s response to the work was inhibited by its conceptualisation of ‘mental deficiency’, Tizard and his colleagues extended the research through the 1960s, including community homes for severely handicapped children and adults. It should be noted here that the National Society for Mental Handicapped Children sponsored Brooklands and other initiatives.^55^ Crossley does not address this SMO, or others such as the Spastics Society and the National Autistic Society, presumably because they do not appear to be directly related to mental health and mental illness. However, all of these groups impacted on both NAMH and the wider psychiatric field.

In any case, the fundamental issues that informed NAMH’s psychotherapeutically inspired principles regarding ‘normal’ child development also informed mental hygienist ideas regarding residential care and treatment for children and young adults already diagnosed ‘emotionally maladjusted’.^56^ Their maladjustment was considered to be largely a product of earlier inappropriate authority relations of care and discipline. Emotional security had been lacking. Consequently their emotional development had been warped and retarded. Therefore these young people were often considered by mental hygienists to require greater toleration and freedom than normal because their relationship with authority had already become distorted and antagonistic. They needed the freedom to ‘regress’ and ‘work through’ these distorted stages of development.

Although the shared principles with NAMH’s report on institutional care for other children were more radically interpreted regarding residential treatment for ‘maladjusted’ children, they can nevertheless be summarised: The child’s experience is, in principle, considered to be central in deciding whether personal relationships and institutional provision are acceptable.^57^ Freedom and choice are *not* seen as the products of training, discipline or treatment. They are intrinsic elements of psychological growth, adjustment and mental health. Rigid hierarchy, regimentation, insensitive authority, and unreflective staff attitudes are considered detrimental to emotional well-being and development. There is an emphasis on promoting open communication. Mental health does not emerge through doing things to passive or reluctant young people.

The mental hygiene movement favoured and indeed sponsored the concept of self-governing communities as particularly applicable to the young people they considered emotionally maladjusted.^58^ As early as the 1920s and 1930s, the mental hygiene movement had supported experiments with self-governing communities. In 1924 an experiment at the Leytonstone Poor Law Homes for Children had significant input from members of the Tavistock Clinic. In the 1930s mental hygienists had also been closely involved with a self-governing community experiment in Essex.^59^ Mental hygienists’ involvement here resulted in later support for the development of the therapeutic community concept. This included the famous Second World War experiment at Northfield hospital for military psychiatric casualties under the auspices of J. R. Rees, the head of the Tavistock Clinic and his ‘Tavi Brigadiers’.^60^ It also included attempts to reform and rejuvenate mental hospitals in the 1950s and 1960s.^61^ Among the latter were T. P. Rees’ influential advocacy of the concept of the therapeutic community at Warlingham Park Hospital, and D. H. Clark’s equally influential development of the concept at Fulbourn. These psychiatrists were closely involved with NAMH, as was Russell Barton, whose concept of ‘institutional neurosis’ had an associated impact.^62^

The extension of such approaches, from children and young people to adults, mirrors the way in which the psychotherapeutic principles on which they were based penetrated throughout all mental hygienist areas of activity. Through the prioritisation of emotionality and its relational components it became a commonplace among post-war mental hygienists that a core feature of *all* mental disorders was a failure to sustain human relationships.^63^ Not only did this call into question environments based on hospital-like regimes for physical illness, it also disrupted those medical models of mental illness that considered it only as an internal disease process.

In child guidance this concentration on emotionality and its relational aspects had paradoxical effects. It was common for interwar mental hygienists in child guidance to attribute maladjustments in children to the faulty behaviour of parents.^64^ But the subtle shift that child guidance signalled, from an overtly moral terminology of correct discipline and training to such medical terminology as patient, diagnosis and treatment, paradoxically entailed a disruption of the straightforward designation of ‘the patient’. This increasingly became understood in terms of maladjustments of the family being ‘projected’ or ‘funnelled’ into the child. This same style of thinking, with its emphasis on ‘projection’ and funnelling’ of symptoms within a relational milieu, became a classic of therapeutic community theorising, and later of anti-psychiatry.

In associated fashion, psychiatric social workers argued that diagnosis and treatment could not in fact be separated.^65^ Others, such as the psychiatrist Jack Kahn, emphasised that diagnostic concepts and pathogenicity were of a relative nature.^66^ Others simply placed less attention on diagnosis. At a NAMH hostel run on self-governing lines, clinical descriptions were considered ‘meaningless for practical purposes’.^67^ Meanwhile, psychiatrists and social workers associated with an early mental hygiene community treatment scheme that included significant numbers of people previously diagnosed psychotic or mentally deficient, maintained that this type of therapeutic approach must resist dealing with people as ‘types’.^68^ These attitudes were often associated with a concern to avoid relationships that were considered authoritarian and therefore anti-therapeutic.^69^ The psychiatrist Martin James, for example, who served on NAMH’s consultant medical panel throughout the 1950s, summed up the 1965 Child Guidance Inter-Clinic Conference by voicing such concerns. He felt that psychologists and doctors were trained to see people as machines, which desensitised them to emotionality, but social workers’ training enhanced sensitivity and therefore avoided authoritarian interventions.^70^

So the principles of care and treatment, founded around the importance of emotional security, associated mental health with the attainment and sustainment of emotionally literate inter-personal relationships. At its heart this contained scepticism towards authority commands and relationships. From this, there developed a critique of prevailing institutional provision, and destabilisation of a medical model based on physical illness. Freedom and choice became increasingly considered part of the means of therapy and not just its intended result. Associated consequences were an emphasis on more open communication, a blurring of the distinction between the pathological and the normal, misgivings about the relevance and impact of diagnostic labels, and some questioning of professional roles and training.

These principles and associated effects are evident across the areas of mental hygienist activity. They served to weaken the movement’s conceptual foundations. But their expression was uneven, and often inconsistent and superficial. This was largely due to the fact that they were in contradiction with the movement’s overall agenda. Under this agenda, mental health was equated with the notion of a rationally marshalled individual, imbued through adequate intellectual capacity and appropriate familial authority relations, with a stable internal authority that readily acquiesced in the authority relations necessary to the community’s balance and progress. An emphasis was placed on differentiation of status and function according to mental ability and the integration of personality. Behaviours associated with ‘social problems’ were largely related to intellectual incapacities or failures of adequate emotional development consequent of inappropriate familial authority relations. Consequently, social, economic and political inequalities were subordinated under this rendition of individual and social ‘mental health’, and indeed often attributed to the same individual and familial issues.

## Mental Hygiene, Anti-Psychiatry and MIND’s Critique of Psychiatry

For Crossley, the anti-psychiatry movement that emerged in the 1960s set in train a ‘paradigm shift in the wider campaigning culture’. The mental hygiene discourse appeared outmoded. NAMH could not shift its stance to the extent of adopting the radical agenda of anti-psychiatry. Instead it became a ‘representative of the civil rights movement/discourse’.^71^ However, I believe that the relevance of anti-psychiatry to MIND is thrown into a new light once placed alongside the mental hygiene agenda and the ‘discursive transformation’ I have described. The mental hygienist agenda was paradigmatic of the ‘psychiatricisation of social problems’ described by Pearson, and its general equation of ‘adjustment’ with mental health a clear target of anti-psychiatry’s ‘politics of socialisation’. As Pearson noted:

When medicine inserts itself into an understanding of social *disorder*, as it does in the medical model of deviance, it expresses an ideology of social order as a *natural phenomenon*. Conformity—rather than being viewed as a *social* accomplishment—is elevated to the status of ‘health’.^72^

But, as I have argued, influential therapeutic principles developed under the mental hygiene movement constituted a ‘discursive transformation’ of the *contents* of care and treatment. These principles are clearly echoed by anti-psychiatry: the emphasis on freedom and choice as part of the means of therapy, the associated attention to ‘open communication’, reduction of rigid hierarchy and regimentation, the denouncing of unreflective staff attitudes, the blurring of distinctions between the pathological and normal, and misgivings about the impact of diagnostic labels.

Yet their emergence under mental hygiene is distinct from anti-psychiatry. Indeed, given that anti-psychiatry revolted against the insertion of the ‘medical model’ into ‘social problems’ it is paradoxical that it was this very intrusion of medical terminology into child guidance which placed the straightforward medical designation of the ‘patient’ in doubt, and along with it the traditional medical understanding of diagnosis.

The discursive transformation that emerged under the mental hygiene movement was in contradiction with the movement’s overall agenda, and weakened its conceptual foundations. In combination with the pressures of anti-psychiatry and wider elements of social critique it contributed to the mental hygiene movement’s collapse. But I show now how the principles of care and treatment that had emerged were continued and extended in an unstable incorporation under MIND. While there were clearly affinities with anti-psychiatry, I show their distinctiveness by highlighting how MIND incorporated and radicalised these principles regarding people then termed ‘mentally handicapped’ (previously ‘mentally deficient’). This is significant because anti-psychiatry paid little attention to ‘mental handicap’.

MIND’s activity under the directorship of Tony Smythe, the ex-head of the National Council for Civil Liberties, has been commonly depicted as a radical break with the organization’s history through the adoption of a civil rights discourse.^73^ As I have noted, anti-psychiatry was highlighted as an important influence. MIND’s approach has generally been characterised as framing the treatment of mentally ill people in terms of the deprivation of liberty.^74^ It has also been portrayed as asserting an associated ‘right to be different’ outside of psychiatric control or coercion.^75^

One clear way into this is by looking at MIND’s 1978 written evidence to the Minister of State for Education and Science on corporal punishment in schools. Written by the psychiatrist Anthony Whitehead and Maurice Rosen, a general practitioner, this began in classic civil libertarian fashion: ‘Everyone has been protected against assault and battery by the state, except the child attending school.’^76^ But its opposition to corporal punishment displays precisely the therapeutic principles that we have seen informed the mental hygiene movement. ‘All children need love and security’ it emphasised. This should ideally come from the child’s parents or substitute parent figures.^77^ But, since children spent such a large part of the week at school, teachers were inevitably an extension of the home and their attitudes significantly affected children. MIND’s evidence noted that corporal punishment was applied for various reasons but especially disobedience, insolence or aggression. It argued that children who behaved in these ways had not ‘developed effective control over their impulses’. They came mainly, it considered, from homes ‘with little love and security’. The children were left feeling un-loved and rejected. They often responded with a ‘swaggering independence’ expressed in anger, aggression and ‘lack of concern for others’, or a ‘passive submission to authority’. Children from loving homes, it argued, had in contrast learned to ‘control their anger and use it constructively.^78^ Given these facts, the memorandum argued, it was vital that the teacher either adopted the parent’s loving role or compensated in some way for those parents who didn’t do this. At school, MIND argued, the, ‘intelligent, healthy, well-adjusted, attractive child tends to receive recognition, but in contrast the intellectually slow, emotionally deprived, or disturbed child gets little, if any, in spite of his or her greater need’.^79^

Clearly MIND’s rights approach did not simply dismiss issues of socialisation in the name of a straightforward civil libertarian call for protection from assault. Clearly, as well, the mental hygiene movement had founded its agenda on just such a view of the family’s personal relations. It had highlighted the need for authority to be tailored to the need for emotional security. And the same polar maladjustments had been posited: passive submission to authority or rebellion against all authority. MIND’s rendition here however is not only of these psychotherapeutic principles as they had become ‘radicalised’ at the level of care and therapy, but also a connection and extension of them with wider social critique. This had the effect of splitting off the discourse of emotionality from the mental hygienist agenda from which it had largely emerged.

For example, MIND promoted a concept of social justice that took the mental hygienist association of delinquency with the working classes and inverted the reduction of it to family disturbances. Family problems were now seen to be exacerbated by social and economic inequalities *and* welfare approaches that ignored these factors.^80^ In its evidence to the Select Committee on Expenditure Examining, the Children and Young Persons Act 1969 MIND criticised the ‘treatment’ model in childcare and social welfare. It remarked that, ‘Treatment implies the presence of an illness but there is no evidence generally available to suggest that the overwhelming majority of deprived or delinquent children are ill’.^81^ This was a rejection of the psychodynamic underpinnings of the casework approach that dominated social work and of the assumption that a consensus existed, characterised by social participation and citizenship, against which ‘social problems’ could be reduced to individual failures of intellectual capacity or faulty familial authority relations leading to individual ‘maladjustments’.

MIND argued that the 1969 Act confused ‘care’ and ‘control’. It criticised the combination of treatment or education with incarceration and punishment, and considered it to be a form of ‘“double think” … detrimental to mental health and to civil liberties’.^82^ However, MIND’s characterisation of some of these detrimental effects is instructive: they reinforced children’s ‘feelings of failure, difference and defiance’. The same principles, that is, which informed its evidence regarding corporal punishment in schools, and which display a strong line of continuity with the psychotherapeutic principles of care and therapy established by the mental hygiene movement.

Indeed MIND was adamant that committal to care rarely promoted either the personal interests or the mental health of children. It closed most of its residential homes. One of the few it retained, MIND’s director Tony Smythe considered to be an ‘exceptionally good school for highly emotionally disturbed young children’. This had opened in 1969 under NAMH. It operated as a therapeutic community, drawing explicit influence from pioneering work associated with the mental hygiene movement. MIND continued to support, and promote, the therapeutic community concept during the 1970s and 1980s.

Concern over the care of people termed ‘mentally handicapped’ (previously ‘mentally deficient’) was a strong component of MIND’s campaigning. This is important in terms of the impact of anti-psychiatry, since it paid scant attention to ‘mental handicap’. Here again, the ‘discursive transformation’ that I have described fed into MIND’s approach. In 1970, at the beginning of its turn to promoting the rights of patients, NAMH had drawn attention to the plight of children in mental handicap hospitals and, for the first time, explicitly drawn attention to the fact that the principles of childcare promoted under the mental hygiene movement had not been applied in mental handicap hospitals.^83^ MIND’s campaigning for people termed mentally handicapped forcefully advocated the kinds of approaches to residential provision proposed by Jack Tizard, along with his colleagues Norma Raynes and Roy King, and put into practice with Albert Kushlick’s work at Wessex.^84^ These were partly legitimated by reference to Russell Barton, who had applied his notion of ‘institutional neurosis’ to mental handicap hospitals.^85^ The effects of institutional provision, such as, ‘block treatment’, ‘social distance’ and ‘depersonalization’, accompanied as they were with a lack of continuity of care and hierarchical authority, continued to be stressed as detrimental to mental health and development.^86^ When Albert Kushlick’s research unit came under funding threat, MIND publicly defended the importance of its work.^87^

People termed mentally handicapped were no longer a race apart with primitive requirements needing accordingly unsophisticated training and care. The dynamics of emotional experience in learning and living were to be central to the education of both staff and clients. Until recently, it had been only mentally handicapped people themselves who had been deemed to need training in socialization so that they could ‘give’ as well as ‘take’ in human relationships.^88^ But now this was reflected back upon the staff. Carers were to be trained in being emotionally sensitive to the inter-personal dynamics with clients and reflect on their own place in these dynamics.^89^

Fully funded community provision was sought by MIND as part of the means of social integration, participation and citizenship.^90^ MIND emphasised that community provisions must avoid setting up ‘mini-institutions’. Hostels should be ‘homely’ and integrated with the general community.^91^ Its 1976 ‘Home from Hospital’ campaign emphasised the need for integrated community care sustaining supportive human relationships. This was not an assertion of a ‘right to be different’ outside of any psychiatric system. On the contrary, in its report *Co-ordination or Chaos?*, MIND accused the government of denying the reality of mental illness.

## Conclusion

It is the issue of authority and its relation to mental health and therapy that substantially informed MIND’s continuities with the mental hygiene movement *and* its break with it. The mental hygienist psychotherapeutic examination of authority and reinterpretation of it in relation to mental health resulted in a ‘discursive transformation’ that was in contradiction with the movement’s overall programme. This transformation did not simply establish a new relation between authority and those subject to it. It placed an emphasis on tailoring authority commands and relations to a perceived need for emotional security and self-expression, in the interests of mental health. This engendered an emphasis on greater freedom and egalitarian relations in care and therapy. Along with a suspicion of doing things to and for reluctant or passive people, this encouraged critiques of institutional care and the prevailing medical model, along with calls for more open communication.

While there are affinities with anti-psychiatry, this discursive transformation in the principles of care and treatment is recognisably distinct. Crossley is surely right that anti-psychiatry was too radical to be adopted by NAMH when the programme of mental hygiene as a movement began to fall apart. But given Crossley’s valuable representation of the interactional and processual nature of SMOs, it is ironic that this view implies only a set of binary choices for NAMH: civil rights or the ‘too radical’ anti-psychiatry. Crossley’s terminology of discursive formation and transformation in fact allows for the kind of examination of mental hygiene that I have presented here. An appreciation of this transformation allows for a more nuanced picture of the relevance of anti-psychiatry to be constructed.

MIND’s approach was not some simple attempt to posit individual liberty against psychiatric control and coercion, nor was it a straight-forward civil rights call for protection from assault. Important strands of its approach were consistent with the discursive transformation I have described. Under MIND, the principles of this discourse were taken up and brought together in unstable coalition with wider elements of social critique. The mental hygienist association of ‘social problems’, such as ‘industrial unrest’, delinquency or truancy, with the working classes—and its reduction of them to disturbances within the family—was thrown off. So too, was its hierarchical societal image associated with intellectual capacity and emotional ‘maturity’. Social problems were now more closely related to social, economic and civil inequalities, and welfare services that disregarded these factors. Freedom and choice were no longer considered simply the aimed for results of treatment. Nor were they now to be retained at the level of therapeutic technologies. They were intrinsic elements of the development of mental health and its maintenance in a much wider sense. Such an unstable coalition could not last. But, no doubt, the interactions and processes of its demise are equally complex.

## Funding

The author would like to thank the Wellcome Trust for the six month research grant between February and August 2016 on which this article is partially based. (200431/Z/15/Z).

## Footnotes

1 NAMH, *Annual Reports 1969/70, 1971/72, 1973/74*. Wellcome Trust Archives and Manuscripts, Mind Archives: SA/MIN/B/80/7/3 Although most often written in capitals the title MIND is not an acronym, nor did it replace the organisation’s official title.

2 Jonathan Toms, *Mental Hygiene and Psychiatry in Modern Britain* (London: Palgrave Macmillan, 2013); Nick Crossley, *Contesting Psychiatry: Social Movements in Mental Health* (London: Routledge, 2006). I also make reference to some of Crossley’s associated articles.

3 Peter Sedgwick, *Psychopolitics* (London, Pluto Press, 1982), 214–15; Nikolas Rose, ‘Unreasonable Rights: Mental Illness and the Limits of the Law’, *Journal of Law and Society*, 1985, 12, 199–218. This was extended and amended as, ‘Law, Rights and Psychiatry’, in Nikolas Rose and Peter Miller, *The Power of Psychiatry* (Cambridge: Polity Press, 1986), 177–213.

4 Lawrence O. Gostin, ‘From a Civil Libertarian to a Sanitarian’, *Journal of Law and Society*, 2007, 34, 594–616, 596–99.

5 C. H. Rolph, *Believe What You Like: What Happened between the Scientologists and the National Association for Mental Health* (London: Deustch, 1973); NAMH, Annual Report 1969–70. SA/MIN/B/80/7/2; Council of Management Minutes throughout 1967–1972. SA/MIN/A/3/1-3. For an overview of anti-psychiatry that includes Scientology, see for example, Nimesh G. Desai, ‘Antipsychiatry: Meeting the Challenge’, *Indian Journal of Psychiatry*, 2005, 47, 185–7.

6 See Andrew Roberts’ site: <http://studymore.org.uk/mpu#MindandMPU> (accessed on 24 October 2018).

7 Geoffrey Pearson, *The Deviant Imagination: Psychiatry, Social Work and Social Change* (London, Macmillan, 1975), 15.

8 Digby Tantam, ‘The Anti-Psychiatry Movement’, in German E. Berrios and Hugh Freeman, eds, *150 Years of Psychiatry, 1841**–**1991* (London: Gaskell, 1991), 333–47, at 344.

9 Crossley, *Contesting Psychiatry*, 16 and 45.

10 As Crossley notes, it originated in the United States during the first decade of the twentieth century. See also, for example, Theresa R. Richardson, *The Century of the Child: The Mental Hygiene Movement and Social Policy in the United States and Canada* (New York: State University Press, 1989).

11 Crossley, ‘Transforming the Mental Health Field: The Early History of the National Association for Mental Health’, *Sociology of Health and Illness*, 1998, 20, 458–88, 484.

12 See, for example, Peter Barham, *Forgotten Lunatics of the Great War* (New Haven, CT: Yale University Press, 2004), ch. 8, 150–64.

13 Crossley, *Contesting Psychiatry*, 90.

14
*Ibid*., 75; Crossley, ‘Transforming the Mental Health Field’, 470.

15 Crossley, *Contesting Psychiatry*, 75.

16
*Ibid*., 94, 97.

17
*Ibid*., 94.

18
*Ibid*., 98.

19
*Ibid*., 96, 122–3.

20
*Ibid*., 136, 138– -9.

21 John Dewey, ‘The New Psychology’, *Andover Review*, 1884, 2, 278–89. Accessed at <http://psychclassics.yorku.ca/Dewey/newpsych.htm> (accessed on 24 October 2018); G. Stanley Hall, ‘The New Psychology’, *Andover Review*, 1885, 3, 120–35. Accessed at <http://psychclassics.yorku.ca/Hall/newpsych.htm> (accessed on 24 October 2018); William McDougall, *An Introduction to Social Psychology* (London: Methuen & Co., 1908).

22 J. R. Rees, *The Health of the Mind* (London: Faber & Faber, 1929); Cyril Burt, Introduction to William McDougall, *Psychology: The Study of Behaviour* (London: Oxford University Press, 1952).

23 Cyril Burt, Preface to Cyril Burt, ed., *How The Mind Works*, 2nd edn (London: George Allen and Unwin, 1945 [1933]), 7.

24 Letitia Fairfield, ‘Crime and Punishment’, *Mental Hygiene*, 1931, 4, 17–20, 18. SA/MIN/B/80/30/1.

25 J. Burnett Rae, ‘Psychotherapy: Its Place and Implications’, *Mental Hygiene Bulletin*, 1931, 3, 1–7, at 3. SA/MIN/B/80/45; Rt. Hon. Sir Lesley Scott, Chairman’s introduction to discussion on, ‘The Proper Care of Defectives Outside Institutions’ in Central Association for Mental Welfare, *Report of a Conference on Mental Welfare Held in the Central Hall, Westminster, London, SW on Thursday and Friday, December the 2^nd^ and 3^rd^, 1926* (London: CAMW, 1926), 17–23, at 21–2.

26 See, for instance, Hugh Crichton Miller, *The New Psychology and the Teacher* (New York: Thomas Seltzer, 1922).

27 Hugh Crichton Miller, ‘Adaptation, Successful and Unsuccessful’, *Postgraduate Medical Journal*, 1926, 1, 60–3, at 62.

28 David Armstrong; *Political Anatomy of the Body: Medical Knowledge in Britain in the Twentieth Century* (Cambridge: Cambridge University Press, 1983), 59.

29 Joseph Schwartz, *Cassandra’s Daughter: A History of Psychoanalysis* (Harmondsworth: Penguin, 1999), 12.

30 Chris Millard, *A History of Self-Harm in Britain: A Genealogy of Cutting and Overdosing* (London: Palgrave Macmillan, 2015). See especially, ch. 2, 62–96.

31 Maurice Craig, ‘Mental Hygiene in Everyday Life’, *Mental Hygiene*, 1933, 7, 57–64, at 63. SA/MIN/B/80/30, 58.

32 R. D. Gillespie, ‘Mental Hygiene as a National Problem’, *Mental Hygiene*, 1931, 4, 1–9, at 9. SA/MIN/B/80/30.

33 Emanuel Miller, ‘The Mind of the Child’, *Mental Hygiene*, 1933, 7, 68–71, at 68. SA/MIN/B/80/30

34 For example, Miller, *The New Psychology and the Teacher*, 36; Mary R Barkas, ‘Guilt’, *Mental Hygiene*, 1932, 6, 29–33, at 33. SA/MIN/B/80/30.

35 Mathew Thomson, ‘Constituting Citizenship: Mental Deficiency, Mental Health and Human Rights in Inter-war Britain’, in Christopher Lawrence and Anna-K. Meyer, eds, *Regenerating England: Science, Medicine and Culture in Inter-War Britain* (Amsterdam: Clio Medica, 2000), 231–50.

36 For example, Maurice Craig, ‘Some Aspects of Education and Training in Relation to Mental Disorder’, *Journal of Mental Science*, 1922, 68, 209–28.

37 Cyril Burt, ‘How the Mind Works in Society’, in Burt, ed., *How the Mind Works*, 179–333, at 240–1 and 245; Hugh Crichton Miller, ‘Educational Ideals and the Destinies of Peoples’, in J. A. Hadfield, ed., *Psychology and Modern Problems* (Plain View, New York: Books for Libraries Press, 1973 [1935]), 131–58, 151.

38 Maurice Craig, ‘Mental Hygiene in Everyday Life’, 62.

39 Gillespie, ‘Mental Hygiene as a National Problem’, 5.

40 C. Flugel, *The Psycho-analytic Study of the Family* (London: The International Psycho-analytical Press, 1921), 120; J. A. Hadfield, ‘Introduction’, in Hadfield, ed., *Psychology and Modern Problems*, 14–15.

41 See Clive Unsworth, *The Politics of Mental Health Legislation* (London: Clarendon Press, 1987), 112–18.

42
*Report of the Royal Commission on Lunacy and Mental Disorder* (Cmd. 2700, 1926), para. 42.

43 Unsworth, *Politics of Mental Health*, 169–70; Doris Odlum, ‘The Meaning of the Mental Treatment Act, 1930’, *Mental Hygiene Bulletin*, 1931, 3, 8–12, at 11–12. SA/MIN/B/80/45.

44 National Council for Mental Hygiene, Ninth Report of the National Council for Mental Hygiene (London: NCMH, 1932). SA/MIN/B/8O/55.

45 W. Paterson Brown, ‘The family neurosis’, in R. G. Gordon, *A Survey of Child Psychiatry* (London: Oxford University Press, 1939), 115–27, 126.

46 Crossley, *Contesting Psychiatry*, 94, 97.

47 Kenneth Soddy, speech on ‘Contributing Factors’ at NAMH, *The Maladjusted Child**—**The Underwood Report and After: Proceedings of a Conference at Church House, Westminster, London on 11^th^ and 12^th^ April, 1957* (London: NAMH, 1957), 23–39, 28. SA/MIN/B/80/43/2.

48 Mary R. Barkas, ‘Guilt’, *Mental Hygiene*, 1932, 29–33. SA/MIN/B/80/30.

49 Denis Martin, ‘The Problem Stated’ in *The Role of Religion in Mental Health: Conference Papers* (London: NAMH, 1967), 7–16, 8. SA/MIN/B/80/43/5.

50 See Jonathan Toms, ‘Mind the Gap: MIND, the Mental Hygiene Movement and the Trapdoor in Measurements of Intellect’, *Journal of Intellectual Disability Research*, 2010, 54, Supp. 1, 16–27. Rare exceptions to this view appear to have existed within and around the mental hygiene movement. One, printed in the CAMW’s *Mental Welfare* is Isabel M. Laird, ‘Some Psychological Problems in “Institutionalising” Defectives’, *Mental Welfare*, 1938, 19, 1–6. Laird, a psychologist at the Royal Scottish National Institution, Larbert, criticised the complete lack of emotionally satisfying relationships with adults available to children sent to mental deficiency institutions. Louise Westwood has described how Helen Boyle, a founder of the NCMH, endorsed the ideals of the Brighton Guardianship Society which resisted ascertainment and segregation in favour of guardianship and foster care in families. See Louise Westwood, ‘A Quiet Revolution in Brighton: Dr Helen Boyle’s Pioneering Approach to Mental Health Care, 1899–1939’, *Social History of Medicine*, 2001, 14, 439–57. Nevertheless, the NCMH annual reports for 1932–36 show that it continued to encourage institutionalisation. SA/MIN/B/8O/55.

51 This was subsequently published by NAMH: Ruth Thomas, *Children Without Homes: How Can They Be Compensated for Loss of Family Life?* (London: NAMH, 1946).

52 H. C. Gunzburg, ‘The Colony and the High-Grade Mental Defective’, *Mental Health*, 1950, 9, 87–92. SA/MIN/B/80/27/10; A. D. B. Clarke and A. M. Clarke, ‘A Rehabilitation Scheme for Certified Mental Defectives’, *Mental Health*, 1954, 14, 4–10. SA/MIN/B/80/27/14.

53 See Toms, *Mental Hygiene and Psychiatry*, 120–25, 130–1.

54 Jack Tizard, *Community Services for the Mentally Handicapped* (London: Oxford University Press, 1964); NAMH, ‘Hospitals—or Family Groups?’, in ‘News and Notes’, *Mental Health*, 1960, 19, 104. SA/MIN/B/80/27/19.

55 See Toms, ‘Citizenship and Learning Disabled People: The Mental Health Charity MIND’s Campaign in Historical Context’, *Medical History*, 2017, 61, 481–99.

56 See, for example, M. W. Hamilton, ‘The Treatment of Children in Hostels’, *Report of Child Guidance Inter-Clinic Conference, 16^th^ November 1946* (London: NAMH, 1946), 24–9. SA/MIN/B/80/54.

57 I say ‘in principle’ because there remained a tension between listening to a child’s *own* expression of its experiences and the *interpretation* of what a child was expressing based on various signs and symptoms. There was nevertheless a clear shift towards more open listening and collaborative living.

58 NAMH, *Committee on Maladjusted Children: Memorandum Submitted by the National Association for Mental Health*, 1952, 11. Robina Addis Archive, Wellcome Trust PP/ADD/J/15/1.

59 See Jonathan Toms, *Mental Hygiene and Psychiatry in Modern Britain* (London: Palgrave MacMillan, 2013), ch. 4, 53–83.

60
*Ibid*., ch. 5, 84–103. Also, Tom Harrison, *Bion, Rickman, Foulkes and the Northfield Experiments: Advancing on a Different Front* (London: Jessica Kingsley, 2000), ch. 5, 152–81.

61 See Toms, *Mental Hygiene and Psychiatry*, ch. 7, 137–71.

62 Recent historical work has usefully examined connections between the development of therapeutic community experiments and anti-psychiatry. However, they do not connect these with the mental hygiene movement. See, Oisín Wall, ‘The Birth and Death of Villa 21’, *History of Psychiatry* 2013, 24, 326–40; Catherine Fussinger, ‘“Therapeutic Community”, Psychiatry’s Reformers and Anti-Psychiatrists: Reconsidering Changes in the Field of Psychiatry after World War Two’, *History of Psychiatry*, 2011, 22, 146–63.

63 H. V. Dicks, ‘Strains Within the Family’, NAMH, *Strain and Stress in Modern Living: Special Opportunities and Responsibilities of Public Authorities: Proceedings of a Conference Held at Friends House, London, N.W.1, 25^th^ to 26^th^ March, 1954* (London: NAMH, 1954), 28–37, 28. SA/MIN/B/80/43/1; T. P. Rees, ‘Back to Moral Treatment and Community Care’, *Journal of Mental Science*, 1957, 103, 431, 303–313. Similar thinking is alluded to in, NAMH, *Questions on Our Minds* [booklet], 2nd edn (London: NAMH, 1966), 5.

64 For example, Gillespie, ‘Mental Hygiene as a National Problem’, 9.

65 Noel K. Hunnybun and Lydia Jacobs, *Interviews with Parents in a Child Guidance Clinic* (London: APSW, 1946), 4.

66 J. H. Kahn, ‘A Wider Concept of Deprivation’, in *Clinical Problems in Children of Primary School Age: The Proceedings of the 19^th^ Child Guidance Inter-Clinic Conference* (London: NAMH, 1963), 5–16, at 7–8. SA/MIN/B/64.

67 David Wills, *Reynolds House: A Report of the First Five Years* (London: NAMH, 1969), 36.

68 T. A. Ratcliffe and E. V. Jones, ‘Intensive Casework in a Community Setting’, *Case Conference*, 1956, 2, 17–23, at 18.

69 NAMH, ‘Memorandum Prepared for the Working Party on Social Workers’, 1956, para. 24. SA/MIN/B/118.

70 Martin James, ‘Summing Up’, *Child Guidance: Function and Social Role: Report of the 21^st^ Child Guidance Inter-Clinic Conference, 1965* (London: NAMH, 1965), 51–2. SA/MIN/B/64.

71 Crossley, *Contesting Psychiatry*, 95.

72 Pearson, *Deviant Imagination*, 48.

73 For example, Kathleen Jones, *Asylums and After: A Revised History of the Mental Health Services: From the Early 18th Century to the 1990’s* (London: The Athlone Press, 1993); Clive Unsworth, *The Politics of Mental Health Legislation* (Oxford: Oxford University Press, 1987).

74 For instance, Kathleen Jones; *Asylums and After*, 203; Nikolas Rose, ‘Unreasonable Rights: Mental Illness and the Limits of the Law’, 202–4.

75 Nikolas Rose, ‘Law, Rights and Psychiatry’, 199.

76 MIND, ‘MIND’s Evidence on Corporal Punishment in Schools’, 1978, 1–2, 1. SA/MIN/B/80/10.

77
*Ibid*., 1.

78
*Ibid*., 1.

79
*Ibid*., 2.

80 John Barter, ‘Children at Risk’, *MIND OUT*, 1975, 9, 6–7; MIND, ‘Children in Trouble’, *MIND OUT*, 1975, 9, 4–6, at 6. SA/MIN/B/80/36/1.

81 MIND, ‘MIND’s Evidence to the Select Committee on Expenditure Examining the Working of the Children and Young Persons Act 1969’, 1975, 1–10, at 8. SA/MIN/B/80/10.

82
*Ibid*., 7.

83 NAMH, Annual Report 1969–70, 4–5. SA/MIN/B/80/7/2.

84 MIND, Editorial, ‘The Issues of Mental Handicap’, *MIND OUT*, 1978, 28, 2. SA/MIN/B/80/36/2; Lindsay Knight, ‘Better Services in Wessex?’, *MIND OUT*, 1978, 28, 6–8, 6. SA/MIN/B/80/36/2.

85 Russell Barton, ‘The Institutional Mind and the Subnormal Mind’ in, H. C. Gunzburg, ed., *Advances in the Care of the Mentally Handicapped* (Birmingham: BSSMH, 1973), 13–20.

86 Lindasy Knight, ‘Better Services’, 7.

87 ‘Wessex Research Unit Threatened with Closure’, *MIND OUT*, 1981, 45, 4; ‘Threat to Wessex Research Unit Postponed’, *MIND OUT*, 1981, 46, 4. SA/MIN/B/80/3.

88 H. C. Gunzburg, *Junior Training Centres: An Outline of the Principles and Practices of Social Education and Training of the Mentally Subnormal Child* (London: NAMH, 1963) [reprinted 1966], 10.

89 MIND, *The Mental Handicap Component in Social Work Training: A Handbook for Tutors and Training Officers*: *Report of a MIND Working Party* (London: MIND, May 1977), 18 (and throughout); Ann Shearer, ‘Institutions Don’t Have to Be Bad’, *MIND OUT*, 1981, 50, 11–13, 13. SA/MIN/B/80/3.

90 Larry O. Gostin, *A Human Condition*: The Mental Health Act from 1959–1975, Observations, Analysis and Proposals for Reform (London: MIND, 1975), 13–14; MIND, *Services for Mentally Handicapped People: MIND’s Evidence to the Royal Commission on the National Health Service* (London: MIND, 1977).

91 Alison Wertheimer, ‘Co-ordination or Chaos—The Rundown of Psychiatric Hosptials’, *Royal Society of Health Journal*, 1975, 95, 3, 136–8, 137–8.

## Notes

Jonathan Toms is an Honorary Research Fellow in the Department of History at the University of East Anglia.

